# Does the cognitive architecture of simplex and multiplex ASD families differ?

**DOI:** 10.1007/s10803-015-2572-9

**Published:** 2015-09-04

**Authors:** Anoek M. Oerlemans, Catharina A. Hartman, Barbara Franke, Jan K. Buitelaar, Nanda N. J. Rommelse

**Affiliations:** Department of Cognitive Neuroscience, Donders Institute for Brain, Cognition and Behaviour, Radboud university medical center, Nijmegen, The Netherlands; Karakter Child and Adolescent Psychiatry University Centre, Reinier Postlaan 12, 6525 GC Nijmegen, The Netherlands; University of Groningen, University Medical Center Groningen, Groningen, The Netherlands; Department of Human Genetics, Radboud university medical center, Nijmegen, The Netherlands; Department of Psychiatry, Donders Institute for Brain, Cognition and Behaviour, Radboud university medical center, Nijmegen, The Netherlands

**Keywords:** Autism Spectrum Disorder (ASD), Simplex-multiplex stratification, Family, Unaffected siblings, Cognition

## Abstract

Children with an autism spectrum disorder (ASD) and their unaffected siblings from 54 simplex (SPX, one individual in the family affected) and 59 multiplex (MPX, two or more individuals affected) families, and 124 controls were assessed on intelligence, social cognition and executive functions. SPX and MPX ASD probands displayed similar cognitive profiles, but within-family contrasts were highest in SPX families, suggesting SPX-MPX stratification may help parse etiological heterogeneity of ASD. Unaffected siblings (regardless SPX or MPX) were mostly unimpaired, suggesting that cognitive problems may be part of the defining features of ASD, rather than being an endophenotypic trait. Except for affective prosody, which appeared to be the most sensitive cognitive marker for detecting familial risk for ASD.

**Electronic supplementary material:**

The online version of this article (doi:10.1007/s10803-015-2572-9) contains supplementary material, which is available to authorized users.

## Introduction

Autism spectrum disorder (ASD) is a group of highly heritable and severely impairing neurodevelopmental disorders, characterized by impairments in social interaction and communication, and restricted, stereotyped and repetitive behaviour (American Psychological Association 2013). ASD is the most heritable of all complex neuropsychiatric conditions, with heritability estimates ranging up to 90 % (Lichtenstein et al. 2010). ASD is marked by substantial heterogeneity in symptom presentation, developmental course and etiologic mechanisms (Jones and Klin 2009). The genetics of ASD is complex with involvement of both rare and common genetic variants. Rare genetic variants predisposing to ASD are currently thought to account for 10–20 % of all ASD cases (Betancur 2011). They include rare mutations in genes which lead to monogenic disorders that are frequently associated with ASD, such as fragile X syndrome and tuberous sclerosis, as well as mutations and copy number variations (CNVs, these constitute deletions or duplications of larger fragments of DNA often involving several genes) that may contribute to (mono- and) oligogenic forms of ASD (Betancur 2011 Persico and Napolioni 2013). Common variants, e.g. single nucleotide polymorphism (SNPs), implicated in the etiology of ASD, on the other hand, are assumed to each contribute a (very) small increase in disease risk (Wang et al. 2009). As ASD strongly reduces reproductive fitness, it has been argued that part of the genetic contribution to ASD is due to *de novo* mutations (Neale et al. 2012; D’Onofrio et al. 2014). In addition to the strong genetic background, environmental influences, gene × environment interaction, epigenetic factors, and pre-perinatal complications also play an important role in susceptibility to ASD (Gardener et al. 2009, 2011; Dietert et al. 2011; Kinney et al. 2010; Wong et al. 2014). Multiple causal pathways may thus underlie the same clinical profiles, and, at the same time, the complex etiology may result in highly heterogeneous clinical profiles.

The heterogeneous character of ASD strongly hinders research into etiology and effective treatment. An approach to parse etiologic heterogeneity is to form more homogeneous subgroups of patients based on the familial occurrence of the disorder. Several studies have reported on the genetic differences between families with only one individual with ASD (the so-called single-incidence or simplex [SPX] families) compared to families with two or more affected individuals (multiple-incidence or multiplex [MPX] families). These studies reported a more than threefold rate of de novo mutations in SPX families (~7–10 %), compared to MPX families (~2–3 %) or control families (~ 1 %) (Sebat et al. 2007; Marshall et al. 2008). In MPX families, shared genetic predispositions based on a multifactorial etiology of common genes appear to play a more important role (Freitag 2007), with members of MPX families more often exhibiting ASD traits compared to members of SPX families (Virkud et al. 2009; Gerdts et al. 2013). We recently replicated the latter finding in the sample described in the current study (Oerlemans et al. 2015). These findings suggest that individuals from SPX families are more likely than individuals from MPX families to develop ASD as a result of sporadic genetic and/or non-genetic causes strictly personal to the patient.

Assuming that SPX-MPX stratification identifies forms of ASD with a different genetic architecture, we aimed to study whether cognitive deficits differ between SPX and MPX families, in probands and/or in unaffected siblings. Previous studies in individuals with ASD have found deficits in intelligence (typically strengths in performance IQ (PIQ) over verbal IQ (VIQ)), social cognition (SC), executive functions (EF) and central coherence (CC) (Joseph et al. 2002; Black et al. 2009; Happe and Ronald 2008; Pellicano 2012). Direct comparisons of cognitive deficits between individuals with SPX and MPX ASD are mostly lacking thus far, and the vast majority of cognitive studies have failed to clearly specify or adjust for simplex or multiplex ascertainment process. So far, studies in SPX ASD-only samples report a higher frequency of performance > verbal IQ discrepancy in cases compared to controls (Ankenman et al. 2014), and an altered cortical shape in brain regions that have been implicated in communication, higher order social processes (e.g. empathy and theory of mind), spatial attention, visual processing and face recognition (Dierker et al. 2013). Studies in MPX ASD-only samples report deficits in EF components such as planning and set-shifting, theory of mind, and fluid and crystallized intelligence (Nydén et al. 2011). To our knowledge, only one study has examined the association of SPX versus MPX status with cognitive functioning. Verbal and non-verbal IQ and head circumference [HC; associated with impaired brain connectivity and higher order abilities (Courchesne and Pierce 2005)] were compared between children and adolescents with autism from SPX and MPX families. The authors reported that enlarged HC was related to social deficits in SPX, but not MPX individuals, and that individuals with the lowest nonverbal IQ scores were mostly classified SPX, whereas individuals with a higher than average nonverbal IQ were mostly MPX (Davis et al. 2013). These findings suggest that both SPX and MPX forms of ASD are associated with a wide range of similar disabilities in higher order cognitive processes, but that some cognitive factors may be uniquely related to either SPX or MPX ASD (e.g. lower IQ scores were reported for SPX ASD), and more research is needed to clarify this issue.

Studies reporting on the presence of ASD-related cognitive deficits in first-degree relatives are sparse and report inconsistent findings (Oerlemans et al. 2014; Wong et al. 2006; Gokcen et al. 2009). A possible explanation for these discrepant findings might be that these studies did not differentiate between etiologically different (inherited versus non-inherited) forms of ASD and thus might have investigated relatives with and without familial loading as a mixed group. A recent study using SPX-MPX stratification to examine executive function of the parents of patients with familial versus non-familial (sporadic) schizophrenia confirmed this idea and reported that executive functions were only impaired in parents with a family history of schizophrenia (Erol et al. 2012). Of interest to us is whether similar patterns can also be found in familial (MPX) versus sporadic (SPX) ASD.

To test whether the cognitive architecture underlying SPX and MPX autism families is different and useful for parsing the etiological heterogeneity of ASD, the cognitive performance of ASD probands and unaffected siblings from SPX and MPX families was compared with each other and with healthy controls. We selected cognitive tasks that assess various cognitive domains previously implicated in ASD (Gokcen et al. 2009; Eapen et al. 2013), or have been described as promising cognitive endophenotypes for ASD in previous literature (Oerlemans et al. 2013; Oerlemans et al. 2014; Rommelse et al. 2011). We hypothesized that potentially different forms of ASD might result in dissimilar cognitive profiles in SPX and MPX ASD probands, a finding with implications for treatment. Further, we hypothesized that the within family contrast between probands and unaffected siblings regarding cognitive aspects of the disorder was larger in SPX compared to MPX families as indicated by (mild) cognitive deficits (similar to their affected brother/sister) compared to controls in unaffected siblings from MPX, but not SPX families, a finding highly relevant to the identification of cognitive endophenotypes for genetic research.

## Method

### Participants

ASD families were recruited as part of the large family-genetic Biological Origins of Autism (BOA) study, (as described previously in van Steijn et al. 2012). Case families were recruited through an outpatient clinic specialized in ASD and ADHD pathology (Karakter Child and Adolescent Psychiatry University Centre) and the Dutch Autism Association (NVA). Families potentially satisfying inclusion criteria received an information brochure and, if interested, were asked to return a pre-stamped response card. Control families were recruited from the same geographical regions as the participating case families via information leaflets. Inclusion criteria for all participants were at least two biological siblings (in case families: at least one child with a clinical diagnosis of ASD [Autism, Asperger’s Syndrome (AS) or PDD-NOS (APA 2000); diagnosis mostly based on Autism Diagnostic Interview- Revised (ADI-R) and Autism Diagnostic Observation Scale (ADOS) assessment]) and one biological parent willing to participate, offspring age between 4 and 20 years, European Caucasian descent, and a IQ ≥ 70, and no diagnosis of epilepsy, brain disorders or known genetic disorders, such as Down-syndrome or Fragile-X-syndrome in order to reduce etiological heterogeneity and provide an ASD sample with considerable clinical homogeneity. Selected controls were required to have no formal or suspected ASD.

Further, children were required to have an IQ ≥ 70 for two reasons. First, to ensure that the children were able to perform the cognitive tasks selected in this study. One of the difficulties that plague the literature in general and hinders research in the low-functioning ASD group is that comparable versions of tasks tapping relevant cognitive domains that can be used in lower functioning individuals are not available. Second, studies examining sporadic genetic mutations in ASD have found that that significant signals in ASD (e.g., excess of de novo loss of function mutations, excess of genes with multiple functional de novo mutations) are predominantly found in individuals with ASD combined with low IQ or intellectual disability (ID) (Robinson et al. 2014; Samocha et al. 2014). Robinson and colleagues reported that de novo mutations are present predominantly in male cases with low IQs, whereas boys with ASD who have normal/high IQs have the same number of de novo mutations as do individuals without ASD. However, female cases had a higher frequency of sporadic genetic events across the severity distribution (i.e. both high and low IQs) (Robinson et al. 2014). Less is known about the impact of sporadic mutations in children with ASD with normal to above average IQs. Potentially, the genetic architecture among ASDs varies as a function of IQ. However, not everyone with a de novo mutation has severe symptoms, indicating that one can have the same high-risk genetic mutation in children with IQ > 100 and children on the low end of the IQ spectrum, but that the mutations in the high-IQ individuals have more moderate effects (Ronemus et al. 2014). The focus of our study was to look at the role of sporadic versus common genetic variants in high-functioning individuals with ASD.

Both the children already clinically diagnosed with ASD, their siblings and their parents were carefully phenotyped for ASD using validated and standardized questionnaires and diagnostic interviews. For all children scoring above clinical cut-off (>10 for the parent version or >15 for the teacher version) on the social communication questionnaire (SCQ) (Rutter et al. 2003), a formal diagnosis of ASD was made by a certified researcher using the Autism Diagnostic Interview-Revised (ADI-R) (Le Couteur et al. 2003 (Dutch version: De Jonge and De Bildt 2007). A lower cutoff was chosen for the parent reported SCQ to avoid false negatives in their undiagnosed offspring (van Steijn et al. 2012). Parents were screened with the Autism Spectrum Quotient (AQ) (Baron-Cohen et al. 2001) and the Adult Social Behavior Questionnaire (ASBQ) (Horwitz et al. 2005). Parents scoring above clinical cut-off were considered a suspected case (for more details, see Oerlemans et al. 2014b). All instruments are validated instruments for screening ASD (de Bildt et al. 2013; Hoekstra et al. 2008; Rutter et al. 2003).

Families were then stratified into SPX and MPX based on the number of affected individuals. SPX families were required to have a single-affected proband, a minimum of one male sibling and all siblings and parents of the proband unaffected by ASD. Families were excluded if (a) only one unaffected parent from a presumed SPX family based on number of affected children participated in this study (to minimize the risk of erroneous categorization because of missing parental data) and (b) if the affected proband had only female unaffected siblings [to account for higher sibling recurrence risk in male siblings than female siblings (Robinson et al. 2014; Ronemus et al. 2014)]. Families with siblings and/or parents who displayed (sub) threshold ASD symptoms, in addition to the proband, were categorized as multiplex (MPX). A total of 54 SPX ASD probands (55.6 % firstborn), 77 SPX ASD unaffected siblings, 91 MPX ASD probands (48.4 % firstborn), 46 MPX ASD unaffected siblings, and 124 control children were included in the current sample. SPX and MPX ASD families did not differ from each other on family size and parental educational level, but had a larger family size and lower maternal educational attainment than control families. Boys were overrepresented in both proband groups and in SPX unaffected siblings, but were underrepresented in MPX unaffected siblings and controls. MPX unaffected siblings were slightly older than other groups, see Table 1 for sample characteristics and Supplement Table 1 for a full description of phenotyping and family classification (available online).

**Table 1:** Sample characteristics

	Controls (c)	Simplex		Multiplex		Group contrasts ASD versus controls
		1. Probands	2. Unaffected siblings	3. Probands	4. Unaffected siblings	
	M (sd)	M (sd)	M (sd)	M (sd)	M (sd)	
Number of children^a^	124	54	77	91	46	
Mean number of children/family	2.3	2.7		2.8		SPX = MPX > controls
Education father^b^	4.9 (1.1)	4.6 (1.1)		4.5 (0.8)		SPX = MPX = controls
Education mother^b^	5.0 (0.9)	4.4 (0.9)		4.5 (0.8)		SPX = MPX < controls
Age	10.9 (3.6)	12.3 (3.5)	12.4 (3.6)	11.6 (3.4)	12.0 (3.7)	1 = 2 = 3 = c, 4 > c
Sex (% males)	41.9	85.2	72.7	71.4	41.3	1 = 2 = 3 > 4 = c
Mean estimated total IQ (range)	107.9 (79–136)	100.7 (72–131)	106.6 (71–147)	100.6 (72–133)	104.4 (79–122)	1 = 2 = 3 = 4, 1 = 3 < c, 2 > 4, 2 = c, 4 = c
SCQ Total Score	3.0 (2.6)	17.9 (6.6)	3.2 (3.3)	19.6 (6.5)	6.2 (6.3)	1 = 3 > 4 > 2 = c
CSBQ ASD core^c^	2.6 (3.8)	26.2 (11.4)	5.4 (6.2)	27.5 (8.6)	11.5 (10.1)	1 = 3 > 4 > 2 = c

*ASD* autism spectrum disorders, *SPX* simplex, *MPX* multiplex, *SCQ* social communication questionnaire, *CSBQ* child social behavior questionnaire, *c* controls; 1 = SPX probands; 2 = MPX probands; 3 = SPX unaffected siblings; 4 = MPX unaffected siblings

^a^Affective prosody was not administered to children younger than 9 years of age and therefore based on 42 SPX probands, 70 MPX probands, 62 SPX unaffected siblings, 34 MPX unaffected siblings and 79 controls

^b^Education is the mean education level of fathers and mothers of probands and their unaffected siblings from SPX and MPX ASD families. Educational attainment is rated on a 7-point scale: 1 = nursery school, 2 = primary education, 3 = secondary education first phase (high school), 4 = secondary education, second phase, 5 = higher education first phase (bachelor), 6 = second education second phase (masters), 7 = higher education third phase (PhD)

^c^ASD core is an aggregate score of the CSBQ subscales reduced contact and social interests, difficulties in understanding social information, stereotyped behaviour and fear of and resistance to changes

### Measures

Cognitive functioning was examined across a range of domains. Verbal IQ (VIQ) was prorated by two subtests of the Wechsler Intelligence Scale for Children or Wechsler Adult Intelligence Scale, namely Similarities and Vocabulary. Performance IQ (PIQ) was prorated by Block Design and Picture Completion (Wechsler 2000, 2002). These selected WISC-III subtests are known to correlate between .90 and .95 with the Full-scale IQ (Groth-Marnat 1997). Three social cognition tasks were administered: face recognition, identification of facial emotions, and recognition of affective prosody. Face recognition was measured by asking children to identify a target face in a display set that consisted of four faces. Identification of facial emotions was measured by asking children to judge whether or not the presented photograph of a human face showed one of four target emotions (happiness, sadness, anger, fear). To test the ability to recognize affective prosody, children were instructed to listen through a headphone to (neutral) spoken sentences that were spoken in a happy, sad, angry or frightened manner and verbally identify the emotion in the voice. Four executive function tasks were included: response inhibition, visual and verbal working memory, and set shifting. Response inhibition was measured with the commonly used Go-NoGo paradigm where participants were instructed to withhold a response when the NoGo target was depicted. Visual and verbal working were measured by instructing the participants to correctly reproduce sequences of figures (visual) or digits (verbal) that increased in difficulty after each succeeded trial. Set shifting was measured by administering a task that required a mixture of compatible and incompatible responses, hypothesized to require a higher level of cognitive flexibility. Cognitive tasks were selected from the Amsterdam Neuropsychological Tasks (ANT) program, which is a computer-aided assessment battery that allows for the systematic evaluation of information processing capacities and has been proven to be a sensitive and valid tool in research into autism-related disorders. Test–retest reliability and validity of the ANT-tasks are satisfactory and have been described in De Sonneville (2005). Table 2 provides an overview of the cognitive tasks used. For full task descriptions, see Appendix 1 (available online) or elsewhere (Oerlemans et al. 2013).

**Table 2:** Description of the neuropsychological tasks

Task^a^	Measurement potential	Dependent variables
Intelligence		
Vocabulary, similarities, block design, picture completion of WISC-III/WAIS-III	Estimated IQ	VIQ and PIQ
Social cognition		
Face recognition	Face recognition	Mean reaction time (ms)
Identification of facial emotions	Identification of facial emotional expressions	Mean reaction time (ms)
Prosody	Affective prosody	Mean reaction time (ms)
Executive function		
GoNoGo	Inhibition	Percentage false alarms—percentage misses
Digit span	Verbal working memory	Max span backwards
Spatial temporal span	Visuospatial working memory	Percentage correct identified targets in correct order (part backward)
Response organization objects	Cognitive flexibility	Percentage errors

*WISC/WAIS-III* Wechsler Intelligence Scale for Children or Wechsler Adult Intelligence Scale-III

^a^Details on each of the paradigms are provided elsewhere (Oerlemans et al. 2013)

### Procedure

Cognitive assessment of participants took place at Karakter Child and Adolescent Psychiatry University Centre Nijmegen and is described in more detail elsewhere (Oerlemans et al. 2013). If possible, stimulants were discontinued for at least 24 h before testing and non-stimulants according to guidelines to allow for sufficient wash-out. Children were motivated with small breaks and received a gift at the end of the session. Parents received a gift voucher (minimum worth €20) and travel-related expenses were covered. Additional data collected included blood or saliva samples and behavioral data of all family members. The study was approved by the local medical ethics board and parents and children (12 years and older) signed for informed consent. Children younger than 12 years of age were asked to give their assent for participation.

### Data analyses

Unlike the other tasks, the affective prosody recognition task was not administered to children younger than 9 years of age. The affective prosody recognition data was based on 42 SPX probands, 70 MPX probands, 62 SPX unaffected siblings, 34 MPX unaffected siblings and 79 controls. The percentage of missing data was <5 % for the majority of dependent measures. Exceptions were missing values of 9.4 % for inhibition and 9.9 % for variability of time estimation. Missings were replaced by means of Expectation Maximization (Tabachnick and Fidell 2001). Analyses were carried out with and without expectation maximization, which revealed similar results and conclusions. Results were therefore reported with missing data replaced. To account for the influence of age and sex on neuropsychological performance, we regressed scores for each measure on age and sex and used the unstandardized residuals as dependent variables. Most of the unstandardized residuals were not normally distributed, therefore a van der Waerden transformation was used to normalize the dependent measures (Norusis 1992). This facilitated the comparison between variables since variables were all depicted on the same scale. Several of the dependent variables were mirrored so that the z-scores of all measures had the same meaning: lower z-scores indicated poorer performance (e.g. more errors, slower and more variable responses).

Linear mixed models (LMM) were used to account for the dependency in the data due to inclusion of siblings by estimating a random intercept. Dependent variables were the cognitive measures and group was the independent variable. We contrasted specific groups of interest to answer our research questions. First, two LMM analyses were run—separately for SPX and MPX ASD families- with group defined as ASD probands versus ASD unaffected siblings versus controls to examine whether cognitive deficits were present in SPX and MPX probands and MPX, but not SPX, unaffected siblings. Second, a LMM with group defined as MPX versus SPX probands was run to examine whether potentially different heritable forms of ASD would result in (dis)similar cognitive profiles in ASD patients. Last, a LMM with group defined as SPX versus MPX ASD unaffected siblings to examine whether cognitive performance of first-degree relatives was poorer in MPX compared to SPX families. Furthermore, within family discrepancy scores (estimated mean of proband minus mean of unaffected sibling) in SPX versus MPX families were compared to examine whether within family contrast was higher in SPX than MPX families. A False Discovery Rate (FDR) correction with a q-value setting of 0.05 was applied to control for multiple testing (Benjamini 2010). Given the unequal sample size for MPX and SPX families, emphasis was given to effect sizes next to the *p* values. Effect sizes (Cohen’s d) were calculated to define small (*d* = .20), medium (*d* = .50), and large effects (*d* = .80) (Cohen 1988). All analyses were carried out in SPSS version 20.

## Results

### Cognitive measures sensitive to SPX-MPX stratification

#### Comparing cognitive deficits in SPX and MPX ASD probands

**Fig. 1:**
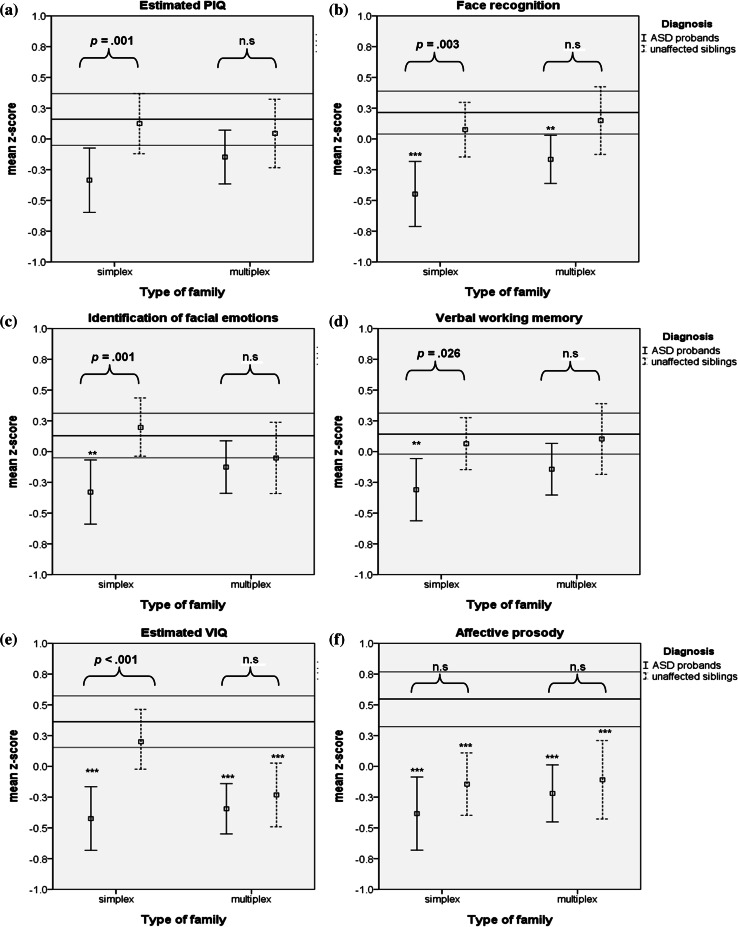
Comparing unaffected siblings from and within-family contrasts in SPX and MPX ASD families. *Note*. *ASD* autism spectrum disorder, *n.s.* non significant. The interpolation lines represent the mean z-score and the 95 % CI of normal controls. The *error bars* represent the 95 % confidence interval (CI). Lower z-scores indicate worse performance. Significant group differences (case groups versus controls) that survived FDR correction are depicted using asterisks (***p < .001, **p < .01). Within-family contrasts are depicted using *squiggly brackets*. Within-family contrasts were higher in SPX compared to MPX families for IQ, emotion recognition and visual working memory, suggesting that affected and unaffected siblings from MPX families resembled each other more closely in cognitive functioning than affected-unaffected siblings from SPX families. Unaffected siblings from both SPX and MPX families were unimpaired on these cognitive domains (**a**–**e**). In *line* with our expectations, we found that MPX unaffected siblings had a significantly lower VIQ (similar to their affected brother/sister) compared to controls, whereas SPX unaffected siblings were unimpaired in this domain. In addition, within-family contrast was highest in SPX ASD families, but non-significant in MPX ASD families for VIQ (**e**). An unexpected finding was that SPX (like MPX) unaffected siblings differed significantly from controls (but not from their affected brother/sister) on affective prosody (**f**)

Testing our first hypothesis, we found that the cognitive profiles of SPX and MPX probands were very similar. Both SPX and MPX probands had significantly lower VIQ (SPX: *p* < .001, effect size in terms of Cohen’s *d* = .69; MPX: *p* < .001, *d* = .68) and PIQ (SPX: *p* = .008, *d* = .42; MPX: *p* = .045, *d* = .28), and poorer face recognition (SPX: *p* < .001, *d* = .65; MPX: *p* = .004, *d* = .40), affective prosody recognition (SPX: *p* < .001, *d* = .92; MPX: *p* < .001, *d* = .70), and verbal working memory (SPX: *p* = .003, *d* = .46; MPX: *p* = .031, *d* = .31) than controls. However, the effects on PIQ and verbal working memory in MPX (but not SPX) probands became non-significant after FDR correction (*q*-values > .10). Further, SPX (but not MPX) probands differed significantly from controls in the identification of facial emotions (SPX: *p* = .010, *d* = .40; MPX: *p* = .097, *d* = .19), suggesting that SPX forms of ASD makes patients more prone to deficits in these domains, see Fig. 1 and Table 3.

**Table 3:** Means and standard errors of the transformed task variables for SPX and MPX probands, their unaffected siblings and normal controls

	Controls (c)		ASD probands	Unaffected siblings	Group contrasts		Within family contrasts		Comparisons between SPX and MPX family members			
									Probands		Unaffected siblings	
	M (se)	Family type	M (se)	M (se)	*p* values*	*d*-values*	*t*	*p*	*p*	*d*	*p*	*d*
VIQ	.36 (.10)	SPX	−.37 (.13)	.22 (.12)	**<.001**/.392**/<** **.001**	.69/.13/.59	**2.56**	**.012**	.930	.02	**.011**	**.47**
		MPX	−.35 (.10)	−.24 (.13)	**<.001/<** **.001**/.409	.68/.57/.12						
PIQ	.15 (.10)	SPX	−.30 (.13)	.12 (.12)	**.008**/.841/**.003**	.42/.03/.42	1.02	.311	.418	.15	.848	.05
		MPX	−.15 (.11)	.07 (.14)	.045/.620/.125	.28/.08/.22						
Face recognition	.22 (.09)	SPX	−.42 (.13)	.08 (.11)	**<.001**/.315/**.004**	.65/.14/.52	1.29	.203	.149	.26	.893	.02
		MPX	−.17 (.10)	.10 (.14)	**.004**/.471/.128	.40/.12/.29						
Identification of facial emotions	.12 (.10)	SPX	−.30 (.13)	.20 (.12)	**.010**/.622/**.002**	.40/.07/.50	**2.38**	**.019**	.305	.14	.102	.27
		MPX	−.12 (.15)	−.08 (.15)	.097/.254/.806	.19/.18/.03						
Affective prosody	.51 (.11)	SPX	−.38 (.15)	−.13 (.13)	**<.001/<** **.001**/.180	.92/.65/.25	.88	.379	.282	.20	.812	.02
		MPX	−.18 (.12)	−.11 (.16)	**<.001/.002**/.706	.70/.65/.07						
Inhibition	.12 (.09)	SPX	−.18 (.13)	.05 (.12)	.066/.655/.160	.31/.07/.23	.53	.596	.623	.10	.941	.01
		MPX	−.09 (.10)	.04 (.14)	.137/.639/.431	.21/.08.14						
Verbal WM	.16 (.09)	SPX	−.29 (.13)	.05 (.11)	**.003**/.428/.039	.46/.11/.36	.48	.624	.431	.13	.592	.04
		MPX	−.16 (.11)	.09 (.15)	.031/.711/.143	.31/.07/.24						
Visual WM	.00 (.09)	SPX	−.21 (.13)	.16 (.11)	.171/.274/**.020**	.21/.16/.39	.16	.873	.727	.07	.655	.04
		MPX	−.14 (.11)	.20 (.15)	.341/.267/.043	.14/.20/.33						
Set shifting % errors	.09 (.09)	SPX	−.09 (.13)	.08 (.11)	.244/.907/.314	.18/.01/.18	.21	.832	.736	.06	.958	.02
		MPX	−.15 (.11)	.06 (.15)	.088/.854/.260	.24/.03/.20						

*ASD* autism spectrum disorders, *SPX* simplex, *MPX* multiplex, *M* mean, *se* standard error, *WM* working memory. Significant group contrasts that survived FDR correction are presented in bold

* *p* values and effect sizes in terms of Cohen’s d (*d* values) are presented in the following order: probands versus controls/siblings versus controls/probands versus siblings. Lower mean scores represent poorer performance

#### Comparing unaffected siblings from SPX and MPX ASD families

In agreement with our second hypothesis, we found that unaffected siblings from MPX families had a significantly lower VIQ (similar to their affected brother/sister) compared to controls (siblings vs. controls: *p* < .001, *d* = .57; siblings vs. probands: *p* = .409, *d* = .12), whereas SPX unaffected siblings were unimpaired in this domain (*p* = .392, *d* = .13). SPX and MPX unaffected siblings also differed significantly from each other on this measure (*p* = .011, *d* = .47). Opposing our hypothesis, both SPX and MPX unaffected siblings scored significantly worse than controls, but similar to their affected brother or sister on affective prosody (SPX: *p* < .001, *d* = .65; MPX: *p* = .002, *d* = .65), see Fig. 1. The unaffected siblings from both SPX and MPX families did not differ from controls on all other cognitive measures (SPX: all *p* values >.27, all *d* values <.16; MPX: all *p* values >.25, all *d* values <.20).

#### Comparing affected and unaffected siblings within SPX and MPX ASD families

Comparing siblings within families revealed that affected and unaffected siblings from MPX families resembled each other more closely in cognitive functioning than affected-unaffected siblings from SPX families. That is, in SPX families, within-family discrepancy (proband-unaffected sibling contrast) was larger for SPX than for MPX families for VIQ (t = 2.56, *p* = .012) and identification of facial emotions (t = 2.38, *p* = .019). SPX probands differed significantly from their unaffected siblings on both measures (VIQ: *p* < .001, *d* = .59; facial emotions: *p* = .002, *d* = .50), whereas MPX unaffected siblings formed an intermediate group, not differing significantly from their affected brothers and sisters on the one hand (*p* values >.12, all *d* values = .03–.29) and from controls on the other hand. This may further suggest that impairments in these cognitive domains are more pronounced in SPX than MPX cases. Significant differences between SPX affected and unaffected siblings were also found for PIQ (*p* = .003, *d* = .42), face recognition (*p* = .004, *d* = .52) and verbal working memory (*p* = .039, *d* = .36), although the latter effect became non-significant after FDR correction (corrected *p* = .07). For visual working memory, significant affected-unaffected sibling contrasts were found for both SPX (*p* = .020, *d* = .39) and MPX (*p* = .043, *d* = .33) families, but, the effect in MPX families did not survive FDR correction (corrected *p* = .15). These findings support the hypothesis that MPX, but not so much SPX, unaffected siblings share some of the ASD-related cognitive deficits.

### Measures not sensitive to SPX-MPX stratification

As describe above, both MPX and SPX unaffected siblings differed significantly from controls (but not from their affected brother/sister) on affective prosody. Further, SPX and MPX probands and unaffected siblings were unimpaired on visual working memory (*p* values >.17, all *d* values <.21), inhibition (*p* values >.07, all *d* values <.31), and set shifting (*p* values >.09, all *d* values <.20), see Table 3. For means and standard errors of the untransformed score of SPX and MPX probands, siblings and controls, see Supplementary Table 2.

## Discussion

The main goal of the current study was to examine whether the cognitive architecture underlying SPX and MPX autism families is different and useful for parsing etiological heterogeneity of ASD. This model of different etiologies in SPX and MPX families is based on evidence from behaviorally-based and genetic research (Sebat et al. 2007; Marshall et al. 2008; Freitag 2007; Virkud et al. 2009; Gerdts et al. 2013). We hypothesized that (a) the different forms of ASD might result in dissimilar cognitive profiles in SPX and MPX ASD probands, and (b) unaffected siblings from MPX but not SPX would display (mild) cognitive deficits compared to controls. Our results showed that directly comparing SPX and MPX ASD cases, no significant differences were detected and both were associated with impairments in VIQ, PIQ, face recognition, affective prosody recognition, and verbal working memory compared to healthy controls. However, when compared to their unaffected siblings, impairments in identification of facial emotions, VIQ, PIQ, and verbal working memory were more pronounced in SPX cases than MPX cases. Unaffected siblings from MPX families had a significantly lower VIQ (similar to their affected brother/sister) compared to controls, whereas SPX unaffected siblings were unimpaired in this domain. Both MPX and SPX unaffected siblings differed significantly from controls on affective prosody and were unimpaired on the other cognitive domains. ASD probands and unaffected siblings from MPX families resembled each other more closely in cognitive functioning than affected-unaffected siblings from SPX families.

Results support the hypothesis that a partly different cognitive architecture may underlie SPX and MPX forms of ASD, which only becomes evident when contrasting cognitive performances within families. That is, the direct comparison between autistic children from SPX and MPX families revealed very similar cognitive problems, but when using unaffected siblings as an ideal reference group (viewed as indexing the ‘full potential’ of children with ASD had they not developed the disorder and correcting for shared environmental factors), SPX probands seem to be more impaired in intelligence, verbal working memory and emotion recognition than MPX probands, which is not explained by a more severe ASD phenotype in SPX probands (i.e., in our sample, SPX and MPX ASD probands demonstrate equally severe ASD traits, see sample characteristics and Oerlemans et al. 2015). This could indicate that partly different developmental pathways may result in a similar phenotype and similar cognitive deficits, a phenomenon that has been referred to in developmental psychopathology as *equifinality* (Cicchetti and Rogosch 1996). ASD has often been associated with lower full scale IQ or intellectual disability (ID) (Charman et al. 2011). One model that has been proposed for the overlap between ID and ASD suggests that rare, highly penetrant mutations set the stage for abnormal developmental trajectories including ASD, developmental delay and mental retardation (Eapen 2011). Assuming that SPX ASD is more likely than MPX ASD to develop as a result of such rare (sporadic) genetic causes, our finding that SPX probands seem to be more impaired in intelligence than MPX probands and the finding of Davis et al. (2013) that ASD children with low(ered) intelligence levels more often had SPX than MPX forms of ASD corroborate this theory.

SPX unaffected siblings were largely unimpaired on cognitive measures compared to controls, except for affective prosody, whereas MPX unaffected siblings were impaired on both affective prosody and VIQ. Several implications may result from this finding. First of all, it suggests that affective prosody is the most sensitive cognitive marker for detecting familial risk for ASD. This finding is in line with previous analyses using the same cognitive task in a younger subsample of this cohort (Oerlemans et al. 2014). The perception of emotional expressions via affective prosody is highly relevant for the development of Theory of Mind (ToM), which refers to the ability to understand other people’s thoughts, beliefs, and other internal states (Korkmaz 2011). Many believe that social cognition deficits are central to explaining the difficulties experienced by people with ASD (Korkmaz 2011; Baron-Cohen 1995). Our finding that unaffected siblings (regardless SPX/MPX status) were impaired on affective prosody, but not on other cognitive domains, might suggest that impaired social cognition is the primary cognitive deficit in ASD, resulting from shared (genetic and/or environmental) risk factors that disrupt the ability to process emotional cues in individuals with autism and (to some extent) their unaffected first-degree relatives. Second, it suggests that the unaffected siblings from SPX families are not completely free from cognitive deficits. The finding is consistent with findings that although *de novo* genetic variations most likely play a role in the development of simplex ASD, they do not fully explain genetic etiology (Krumm et al. 2013). In other words, also in SPX ASD families some risks may be shared between family members (Klei et al. 2012), and the distinction between MPX and SPX ASD may reflect variation in the magnitude of effects rather than qualitative differences between groups. Third, only a few comparisons between MPX unaffected siblings and controls reached significance. This finding clearly contrasts with studies in ADHD that firmly demonstrate significant impairments on cognitive functions and brain morphology in first-degree unaffected relatives who are at risk of the disorder (Allen et al. 2009; Rommelse et al. 2011). This does not seem to be due to a simple lack of power: visual inspection of the data indicate no or only very minor cognitive impairments on several domains that are impaired in the MPX probands (face recognition, PIQ, verbal working memory). This suggests that—in contrast to ADHD—cognitive factors in ASD may have a stronger determining effect on the development of the final phenotype. That is, cognitive problems may be part of the defining features of ASD, rather than being an endophenotypic trait that can be seen in unaffected relatives. An important exception is affective prosody, suggesting this domain may be sensitive towards familial risk factors for ASD.

Some limitations to this study need to be acknowledged when interpreting the results. First, sample sizes were moderate; it follows that our study needs replication in larger samples to fully uncover effects. Second, reproductive stoppage is a factor in ASD. Stoppage is the phenomenon in which parents who already have a child affected with ASD may decide to not have more children after symptoms appear and/or an affected child receives the diagnosis (Hoffmann et al. 2014). It is difficult to tell if SPX families would be MPX families if not for stoppage, especially when the proband is severely affected. The issue of stoppage also has implications for recurrence risk estimates and birth order studies in ASD (Wood et al. 2014). Ideally, one would account for stoppage, for example by only examining families with unaffected siblings born before the affected probands or by only including information from the first ASD case in each family. Due to our limited sample size, we were unable to do so, but we believe that insofar this has affected our results, it would likely lead to an underestimation of potential differences between SPX and MPX ASD cases. Of note, the family size of the SPX and MPX families did not differ from each other and were slightly larger than control families and in about half of the SPX families an unaffected sibling (29 male, 7 female) was born before the affected child. Third, boys were overrepresented in both proband groups and in SPX unaffected siblings, but were underrepresented in MPX unaffected siblings and controls. This was likely due to the fact that a) ASD is more frequently diagnosed in males and b) because the presence of male unaffected siblings was only required for SPX, but not MPX families. However, we do not believe that this has affected the results, since the effect of sex was controlled for in this study. Fourth, although effort was made to include several tasks tapping the domains of SC and EF, we were not able to assess all aspects of these cognitive domains. For example, fluency, planning and theory of mind were not assessed here. We cannot rule out the possibility that the cognitive functions not studied here are sensitive to familial effects. Fifth, only Dutch participants of European Caucasian ethnicity were included in our study. This may limit the generalization of our findings to other ethnic groups. Also, by focusing on average and high-functioning ASD, the generalizability of our findings to the broad range of ASD is limited. Future research should consider extending these findings to lower-functioning individuals with ASD—a group that is greatly under-represented in research studies—which may reveal different SPX-MPX ratios and more pronounced impairments on the various cognitive domains. Last, the difficulty with matching non-ASD IQ levels with ASD IQ levels should be discussed. Often, studies match cases and controls on mental age or IQ but given that IQ is inherently confounded with ASD, it cannot be fully separated from the effects of the condition (Jarrold and Brock 2004; Dennis et al. 2009). Matching IQ to controls in children with ASD may create unrepresentative groups, with either the ASD group having higher IQs than the population with the disorder, or the control group having IQs below normal expectations (Dennis et al. 2009). The authors suggest that instead, controlling for sociodemographic characteristics may be desirable. Given that estimated verbal and performance IQ (separately) were outcome measures in our study, we did not match on total IQ, except from the inclusion criterion of IQ ≥ 70 for all participants. Sample characteristics reveal that IQ distributions were highly similar for affected and unaffected siblings and controls (albeit higher mean IQ for controls than affected children) and parental educational levels were largely similar across groups, indicating that the controls may be an adequate comparison group. Differences in age and sex across groups were controlled for in the analyses.

All in all, results suggest that some differences between SPX and MPX forms of ASD exist, which becomes evident when contrasting cognitive performances *within* families. These findings may help parse etiological heterogeneity of ASD by stratifying ASD families into families with stronger versus weaker familial aggregation of ASD-related cognitive deficits.

## Electronic supplementary material

Supplementary material 1 (PDF 126 kb)

Supplementary material 2 (PDF 26 kb)
