# Supplementary material for: Does the cognitive architecture of simplex and multiplex ASD families differ?
Source: J Autism Dev Disord. 2015 Sep 4;46:489–501. doi: 10.1007/s10803-015-2572-9 (PMC4724369; doi:10.1007/s10803-015-2572-9)
Supplement: Supplementary file 1 — Supplementary material 1 (PDF 126 kb) [file 10803_2015_2572_MOESM1_ESM.pdf]

SUPPLEMENT TABLE 1. Details on child and parent phenotyping and family classification

Child	The exact screening procedures and measures for ASD phenotyping in children have been described in previous publications which can be consulted for greater detail (van Steijn et al. 2012). The parent and teacher Social Communication Questionnaire (SCQ) (Rutter et al. 2003) and the Child Social Behavior Questionnaire (CSBQ) (Hartman et al. 2006) were used to identify children with ASD symptoms. These questionnaires are validated instruments to measure ASD traits (Rutter et al. 2003; Charman et al. 2007). All children scoring above cut-off on any of the questionnaires underwent full diagnostic ASD assessment, including the Autism Diagnostic Interview-Revised (ADI-R) (Le Couteur et al. 2003). Control children were required to obtain non-clinical scores in order to be accepted in the study.
Parent	Case and control parents were screened for ASD using the Autism Spectrum Quotient (AQ) (Baron-Cohen et al. 2001) and the Adult Social Behavior Questionnaire (ASBQ) (Horwitz et al. 2005; Horwitz et al. submitted). The ASBQ is the adult version of the CSBQ and, although still under development, shows first promising results in terms of reliability and validity for the ASBQ (Horwitz et al. submitted). Parents scoring above cut-off (Hoekstra et al. 2008) on any of the questionnaires, were considered affected**.** Control parents were required to obtain non-clinical scores in order to be accepted in the study.
Family classification	Families were then stratified into SPX and MPX families. SPX families were required to have a single-affected proband, a minimum of one male sibling and all siblings and parents of the proband unaffected by ASD on the basis of non-clinical scores on the screenings questionnaires and/or administered diagnostic interviews. Families with siblings and/or parents who displayed (sub) threshold ASD symptoms, in addition to the proband, were categorized as multiplex (MPX). Families were excluded if a) only one unaffected parent from a presumed SPX family based on number of affected children participated in this study (to minimize the risk of erroneous categorization because of missing parental data) and b) if the affected proband had only female unaffected siblings (to account for higher sibling recurrence risk in male siblings than female siblings).

Note. ASD = autism spectrum disorders; SPX = simplex; MPX = multiplex

References

Baron-Cohen, S., Wheelwright, S., Skinner, R., Martin, J., & Clubley, E. (2001). The autism-spectrum quotient (AQ): evidence from Asperger syndrome/high-functioning autism, males and females, scientists and mathematicians. *Journal of Autism and Developmental Disorders,* 31(1), 5-17.

Charman, T., Baird, G., Simonoff, E., Loucas, T., Chandler, S., Meldrum, D., et al. (2007). Efficacy of three screening instruments in the identification of autistic-spectrum disorders. *British Journal of Psychiatry,* 191, 554-559

Hartman, C. A., Luteijn, E., Serra, M., & Minderaa, R. (2006). Refinement of the Children's Social Behavior Questionnaire (CSBQ): an instrument that describes the diverse problems seen in milder forms of PDD. *Journal of Autism and Developmental Disorders,* 36(3), 325-342

Hoekstra, R. A., Bartels, M., Cath, D. C., & Boomsma, D. I. (2008). Factor structure, reliability and criterion validity of the Autism-Spectrum Quotient (AQ): a study in Dutch population and patient groups. *Journal of Autism and Developmental Disorders,* 38(8), 1555-1566

Horwitz, E. H., Schoevers, R. A., Ketelaars, C. E. J., Kan, C. C., Van Lammeren, A. M. D. N., Meesters, Y., et al. (submitted). Autism Spectrum Disorders (ASD) and adults assessed by self and other report: psychometric properties and validity of the Adult Social Behavior Questionnaire (ASBQ).

Horwitz, E. H., Systema, S., Ketelaars, C. E. J., & Wiersma, D. (2005). Twee zelfrapportagescreeningsvragenlijsten voor autismespectrumstoornissen bij volwassen. *Tijdschr Psychiatr,* 47(5), 291-298.

Le Couteur, A., Lord, C., & Rutter, M. (2003). *The Autism Diagnostic Interview—Revised (ADI-R).* Los Angeles: CA: Western Psychological Services.

Rutter, M., Bailey, A., Berument, S., Lecouter, A., Lord, C., & Pickles, A. (2003). *Social Communication Questionnaire (SCQ)*. Los Angeles, CA: Western Psychological Services.

van Steijn, D. J., Richards, J. S., Oerlemans, A. M., de Ruiter, S. W., van Aken, M. A., Franke, B., et al. (2012). The co-occurrence of autism spectrum disorder and attention-deficit/hyperactivity disorder symptoms in parents of children with ASD or ASD with ADHD. *Journal of Child Psychology and Psychiatry and Allied Disciplines*, 53(9), 954-963
