# Supplementary material for: Does the cognitive architecture of simplex and multiplex ASD families differ?
Source: J Autism Dev Disord. 2015 Sep 4;46:489–501. doi: 10.1007/s10803-015-2572-9 (PMC4724369; doi:10.1007/s10803-015-2572-9)
Supplement: Supplementary file 2 — Supplementary material 2 (PDF 26 kb) [file 10803_2015_2572_MOESM2_ESM.pdf]

SUPPLEMENTARY TABLE 2. Means and standard errors of the **untransformed** task variables for SPX and MPX probands, their unaffected siblings and normal controls (corrected for age and sex)

		Controls (c)		ASD probands	unaffected siblings
		M (se)	Family type	M (se)	M (se)
VIQ		109.3 (1.8)	SPX	93.5 (2.3)	105.5 (21)
			MPX	96.4 (2.0)	99.0 (2.5)
PIQ		108.2 (1.9)	SPX	98.9 (2.4)	107.0 (2.2)
			MPX	102.5 (2.1)	105.8 (2.6)
Face recognition	Mean reaction time (ms)[a]	17734.8 (42.9)	SPX	2111.1 (66.8)	1830.1 (55.2)
			MPX	1938.2 (50.6)	1770.0 (69.6)
Identification of facial emotions	Mean reaction time (ms)[a]	969.4 (18.4)	SPX	1073.3 (26.0)	964.7 (22.9)
			MPX	1019.0 (20.9)	1003.6 (28.0)
Affective prosody	Mean reaction time (ms)[a]	2949.3 (63.9)	SPX	3539.1 (84.9)	3384.2 (72.6)
			MPX	3424.6 (68.4)	3321.7 (93.3)
Inhibition	Accuracy (% false alarms-% misses)[b]	20.5 (1.4)	SPX	25.8 (2.1)	22.0 (1.8)
			MPX	24.1 (1.7)	21.2 (2.3)
Verbal WM	Max. span backwards[c]	4.2 (0.1)	SPX	3.7 (0.2)	4.1 (0.1)
			MPX	3.8 (0.1)	4.2 (0.2)
Visual WM	% correct identified targets[d]	56.2 (2.1)	SPX	51.2 (2.9)	58.8 (2.6)
			MPX	52.2 (2.3)	59.8 (3.1)
Set shifting % errors	Accuracy (% errors)[e]	7.0 (0.7)	SPX	8.6 (1.1)	7.3 (0.9)
			MPX	9.2 (0.8)	7.7 (1.2)

Note. ASD = autism spectrum disorders, SPX = simplex, MPX = multiplex, M = mean, se = standard error, WM = working memory.

[a]higher scores represent slower reaction times
[b]higher mean score represents less accurate
[c]higher maximum span represents better verbal working memory
[d]higher mean score represent more accurate
[e]higher mean score represents more errors
